# Prescription and Effects of Strength Training in Individuals with Intellectual Disability—A Systematic Review

**DOI:** 10.3390/sports9090125

**Published:** 2021-09-04

**Authors:** Miguel Jacinto, Rafael Oliveira, João P. Brito, Alexandre D. Martins, Rui Matos, José Pedro Ferreira

**Affiliations:** 1 Faculty of Sport Sciences and Physical Education, University of Coimbra, 3040-248 Coimbra, Portugal; jpferreira@fcdef.uc.pt; 2 Life Quality Research Centre (CIEQV), 2040-413 Rio Maior, Portugal; rafaeloliveira@esdrm.ipsantarem.pt (R.O.); jbrito@esdrm.ipsantarem.pt (J.P.B.); alexandremartins@esdrm.ipsantarem.pt (A.D.M.); rui.matos@ipleiria.pt (R.M.); 3 Sports Science School of Rio Maior, Polytechnic Institute of Santarém, 2040-413 Rio Maior, Portugal; 4 Research Center in Sport Sciences, Health Sciences and Human Development, 5001-801 Vila Real, Portugal; 5 School of Education and Social Sciences, Polytechnic Institute of Leiria, 2411-901 Leiria, Portugal

**Keywords:** intellectual disabilities, neuromuscular training, physical exercise program, resistance training

## Abstract

The practice of physical exercise (PE), especially strength training (ST), has health benefits in the healthy population; however, the literature is scarce in the recommendations related to the population with intellectual disability (ID). This study represents the first analysis on the topic and aims to examine the structure and efficacy of ST experimental intervention programs in individuals with ID. This systematic review was carried out between January and April 2021, using the PubMed, Web of Science, Scopus, and SPORTDiscus databases, according to the PRISMA guidelines. From a total of 166 studies, eight were included in the present systematic review. The studies included a total of 280 individuals (18.23 ± 2.86 years old). The main features of the exercise programs are: 12 weeks average duration, three weekly sessions of 45–60 min, six to seven exercises targeting the main muscle groups, two to three sets, 6–12 repetitions, and avoiding free weights for safety reasons. The main results showed increments in strength, balance and fat-free mass and decrements in fat mass and waist circumference. It is a useful guideline for PE technicians to prescribe and adjust correctly in order to not only promote physical fitness, but improve the quality of life of individuals with ID.

## 1. Introduction

The World Health Organization [1] global recommendations have recently highlighted the benefits of physical exercise (PE) for the health of youngsters and adults living with intellectual disabilities (ID). ID is a deficit in cognitive and adaptive functioning, as well as in the conceptual, social, and practical domains diagnosed before 18 years of age. Individuals can be diagnosed with different degrees: mild, moderate, severe, and profound ID [2]. Several studies in the literature report a high incidence of sedentary behavior in people with ID [3,4]. On the other hand, the health risks in these individuals increase when compared to the general population, namely in the higher prevalence of hypertension, obesity, and metabolic syndrome [5,6].

Adults with ID are often referred to as high-cost patients, a designation used as a reference to patients classified in 10% of total annual health care costs [7]. They register a high number of annual hospital visits and need more medical drugs than adults without ID. Given the fact that this population tends to have a disproportionate number of comorbidities compared to apparently healthy adults, their quality of life may be affected [8].

Physical inactivity does not promote physical fitness, which leads to lower levels of muscle strength that may also be associated with musculoskeletal problems and insufficiency of the central nervous system in the activation of motor units and some intrinsic dysfunctional muscle properties, namely atrophies or hypotonia [7]. Since premature physical aging is a characteristic of most individuals with ID, the early loss of muscle mass [8] is a consequence that is associated with impaired functional capacity, mobility, and other comorbidities [9,10]. Sarcopenia negatively affects body composition as well as the rate of basal metabolism [8,11] and the ability to produce strength, which is related to physical dependence, increased number of falls and hospitalizations, lower perceived quality of life, and increased risk of mortality [12,13].

Thus, it is important to increase PE in a structured and planned way, so that the results related to health and physical fitness improvement are effective [14,15].

Several studies reported promising results from the positive effects of exercise on muscle strength and daily life activities in both healthy individuals [16,17] and individuals with clinical conditions [18,19]. However, there is little evidence to support whether strength training (ST) can improve general health in young adults with ID [20]. Muscular strength is critical for adults with an ID to promote their mobility, cardiovascular capacity, and performance of daily living/recreational/vocational activities [20,21,22]. ST interventions can be effective by improving muscle strength in adults with ID, especially when not combined with other forms of exercise [23].

ST aims to provoke adaptations in the skeletal musculature through overloads, providing an increase in the production of muscle strength and activity of glycolytic enzymes, as well as the production of adenosine triphosphate/phosphocreatine and adaptations in the nervous system, in order to increase the recruitment of motor units [24,25]. During ST, the lower and upper limbs move against a resistance provided by gravity, body weight, dumbbells, straps, weighted bars, or exercise machines [26,27,28]. This entire process results in cellular micro-lesions, mainly in the eccentric action phase, activating defense systems such as neutrophils, macrophages, and cytokines, which will generate reactive oxygen and nitrogen species [29]. These micro-sockets are important for the muscle recovery and regeneration process due to the fusion of satellite cells with a main cell, and the induction of protein synthesis metabolism and muscle tissue recovery [30]. Thereafter, ST seems to induce muscle skeletal adaptations as a result of overload, providing an increase in the production of muscular strength and other central nervous system adaptations [24,25].

ST has also demonstrated positive effects on neuromuscular capacity, on daily living performance activities, and on reducing the decline in sarcopenia [31,32].

Usually, a typical ST program for untrained or intermediately trained healthy adults includes the use of all major muscle groups, performing between two to four sets of 8–10 exercises, for 3–12 repetitions with 2–5 min rest between sets, with a frequency of two to four times per week [33,34]. Meanwhile, a systematic review with meta-analysis found that the effects of training with high loads (i.e., ≥60% of 1 RM or ≤15 RM) compared to low loads (i.e., <60% of 1 RM or >15 RM) and found a similar effect of hypertrophy independent of the loads [24]. Nonetheless, there is a lack of knowledge about ST in people with ID, which reinforces the need verify whether regular ST leads to the beneficial effects apparently observed in the healthy population. Therefore, the purposes of this study are to answer the two following research questions: (i) What are the benefits of ST programs in individuals with ID? ii) What are the most common and effective structural characteristics and guidelines to prescribe ST programs for individuals with ID?

## 2. Methodology

### 2.1. Eligibility Criteria

This systematic review was designed following the items of the PRISMA guidelines [35]. The PICOS strategy [36,37] was defined with the following format: (i) “P” (patients) corresponded to participants with ID (Down syndrome inclusive) of any age, gender, ethnicity, or race; (ii) “I” (intervention) corresponded to an ST program, regardless of the intervention time (ST is considered whenever it is intended to produce muscular tension to overcome any resistance); (iii) “C” (comparison) corresponded to the pre and postintervention comparison or between control group versus intervention group; (iv) “O” (outcome) corresponded to any variable of physical fitness or physiological capacity as a primary or secondary variable under study; (v) “S” (study design) corresponded to randomized controlled trials.

### 2.2. Information Sources and Research Strategies

The systematic search for articles was carried out between January and April 2021, in the following databases: PubMed (all fields), Web of Science, Scopus, and SPORTDiscus (title, abstract, and keywords), considering the period of retreat until 2010. The descriptors used were: “Mental Retardation”, “Intellectual Disability”, “Intellectual Disabilities”, “Down Syndrome”, “Resistance Training”, “Strength Training”, “Neuromuscular Training”, “Resistance Exercise”, “Strength Exercise ”,“ Neuromuscular Exercise ”, with the Boolean operator “ And ” or “ OR ”. Table 1 shows the content of the search.

### 2.3. Inclusion Criteria

For the selection of studies, the following inclusion criteria were considered: (i) randomized controlled trial studies; (ii) intervention studies of any duration; (iii) individuals with ID, regardless of the degree of disability, including those with Down syndrome; (iv) without limitations regarding the number of participants; (v) studies published in English or Portuguese language.

### 2.4. Exclusion Criteria

Likewise, for the selection of studies, the following exclusion criteria were used: (i) studies published before 2010; (ii) studies with participants with another type of disability or pathologies; (iii) articles that do not describe the intervention protocol for ST prescription; (iv) studies where the intervention is focused only is a specific sport; (v) studies in which the intervention is not only ST in the same exercise groups.

### 2.5. Data Extraction Process

After completing the systematic search, duplicates were eliminated and all the articles that did not meet the inclusion criteria were removed. The studies selected in the previous phase were reviewed in their entirety by two independent reviewers (MJ and RO), taking into account the specific eligibility criteria. One of the reviewers (MJ) selected the relevant information about each one of the studies, organized by: authorship, year of publication, country, objectives, participants, type of study, assessment instruments, duration/frequency, type of exercises, intensity, and main results.

After reading the full text of the studies, and according to the eligibility criteria previously defined, the study sample was constituted with eight studies.

### 2.6. Methodological Quality

The PEDro scale, from the Physiotherapy Evidence Database, was used [38], to assess the quality in each study. The scale consists of 11 items, which characterize the different parts of each study. One of the items is not scoreable in the field of sports science (Item 1) and two other items have no applicability (Items 5 and 6). The maximum score on the scale, after being modified, to evaluate the articles is 8 points.

The assessment of the quality in each study was independently estimated by two investigators (MJ and RO) and they were compared and discussed so that there is a consensus.

## 3. Results

### 3.1. Selection of Studies

The initial research carried out in the four databases identified 169 studies. In the first phase, and after reading the titles and the abstracts, 23 potentially relevant studies were identified for the next phase. Considering the inclusion and exclusion criteria, previously defined for this systematic review, of the complete reading of the studies, a sample of nine was constituted for their full analysis.

**Figure 1:**
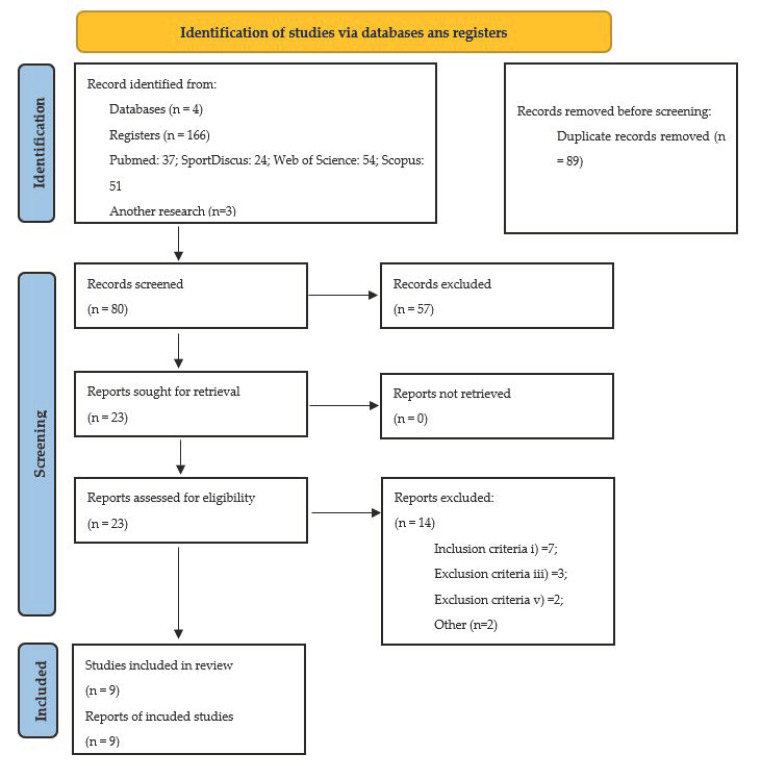
Flow diagram PRISMA.

Figure 1 represents the PRISMA flowchart of this systematic review.

### 3.2. Characteristics of Studies

Table 2 shows the nine studies included for systematic review, as well as the results of the quality of information assessment. The quality analysis of the studies showed that scores varied between six and eight on the PEDro scale, thus presenting a good quality of the methodological procedures.

Table 3 shows the aims, the participants, study designs and the assessment of the instruments/techniques used in the studies included.

### 3.3. Origin

From the nine selected studies that were analyzed in the systematic review, one is from Asia [40], another from Africa [41], two are from Oceania [46,47], two others are from America [42,43] and three are from Europe, with Spain being the country with the largest number of publications about this topic [39,44,45].

### 3.4. Participants

The total number of participants involved in the selected studies are 280, 150 included in the intervention groups and 130 as part of the control groups. The subjects’ mean age from all the studies is 18.23 ± 2.86 years, including children, adolescents, and young adults. All the studies used participants with ID and associated Down syndrome, except for the study of Kachouri et al. [41]. There is a shortage of studies in the literature that implemented ST programs in participants with ID without any other associated condition and using a randomized controlled method.

### 3.5. Evaluation Protocols/Instruments/Techniques

Most studies used assessment instruments, such as the agility test, the “box stacking” test, the “supermarket” test, the “bucket transportation” test, or the stairs up/down test [39,44,46,47] to evaluate functional capacity. The anthropometric assessment was accomplished using weight, height, waist circumference, and Body Mass Index—BMI [42,43]. Some studies also evaluate fat-free mass and fat mass using the electrical bioimpedance method or subcutaneous adiposity skinfolds [42,43,44]. Although there was a wide dispersion in the evaluation methods used in different studies, the neuromuscular capacity is always assessed either through the exercises prescribed in the training programs or through standard assessments. Maximal and submaximal strength tests were used, such as the one max repetition test—1 RM [46,47], the 8 RM [39,45], the handgrip test measured by manual dynamometer [43,45], and different isokinetic strength tests measured by isokinetic dynamometer [45]. It should be noted that Ghaeeni’s [40] and Neto’s [42] studies did not assess neuromuscular capacity, despite applying ST programs. Table 4 shows the duration/frequency, type of exercises, intensity, and results of the nine selected studies.

### 3.6. Characteristics of the Strength Training Protocols

Table 4 shows the characteristics of the ST protocols. The programs’ duration varied between 8 and 16 weeks, with half the studies prescribing 12 weeks [39,42,44,45]. The weekly frequency varied between two and five times, with a greater number of studies showing a weekly frequency of three sessions [39,40,41,42,44,45]. The training sessions duration varied between 45 and 60 min.

The protocols adopted varied from study to study. Six studies applied circuits in ST machines [39,42,44,45,46,47], one study included exercise performed in two surfaces conditions (firm and foam) [41], one study applied exercises with free weights [45], and one study applied an abdominal workout [40].

All studies showed a positive effect from the different ST protocols, which can be seen in Table 4.

## 4. Discussion

The present systematic review aims to analyze the effects of ST in individuals with ID, based on the characterization of several programs implemented, as well as the identification of mean characteristics for ST prescription programs, namely duration, weekly frequency, appropriate assessment methods, and type of exercises. The following subsections will discuss all main point of the ST programs applied.

### 4.1. Program Duration

As mentioned in the results section, the programs’ durations varied between 8 and 16 weeks, with half the studies prescribing 12 weeks [39,42,44,45]. Short-term intervention programs may be a limitation presented in some studies [39,46. Although all studies have shown several positive results (Table 2), further studies with a longer duration are needed to understand the long-term benefits, as well as the type of ST periodization to be applied in the population with ID. We consider that the duration of the training programs is critical to outwit if there are individuals who do not experience significant improvements following an exercise training intervention. Such individuals are commonly termed “non-responders”. However, recently, many researchers have taken a skeptical view as to whether exercise non-response either exists, or is clinically relevant.

### 4.2. Frequency

The weekly frequency followed the recommendations of the American College of Sports Medicine (ACSM) [48]; once weekly frequency occurred between two and five times. This number of weekly TS sessions allows adaptations that lead to catabolism and consequent protein anabolism, allowing the maintenance or increase in lean mass [24]. Moreover, except for Ortiz-Ortiz et al. [43], all the other studies did not report sessions on consecutive days. It is important to reinforce that even the study of Ortiz-Ortiz et al. [43] improved the BMI (*p* < 0.0001) and skin fold of the calf (*p* = 0.008), which suggests future studies should confirm such results.

### 4.3. Session Duration

The training sessions duration varied between 45 and 60 min and their structure consisted of warm-up, main phase, and a return to calm/cool down, which is in accordance with the ACSM guidelines [48]. However, some studies did not mention the session duration [39,44,45,46], which is a limitation, leaving some doubts about the recovery period that was applied, as well as the replicability of the studies.

### 4.4. Sets

The number of sets per exercise varied between two and six, depending on the progress and training periodization. However, the prescription of two or three sets was more frequent [39,42,44,45,46,47], in accordance with ACSM [48]. Some authors state that, in untrained individuals, both single set and multiple sets produce similar increases in muscle strength of upper and lower limbs; that is, in the early stages, the RT, regardless of number of sets, seems to be effective for improving muscle outcomes [49].

### 4.5. Repetitions

The number of repetitions per set varied between 6 and 30; however, most studies prescribed 6 to 12 repetitions [39,42,44,47]. This number is influenced by the prescription of one RM methods or the use of a number of repetitions by exercise (not one RM). The number of sets and repetitions per exercise can vary depending on volume and intensity [50]. This number follows previous recommendations for healthy people [51].

### 4.6. Intensity

ST programs presented different training intensities according to the different objectives established for the development of strength adaptations (endurance, resistance, power, etc.). The intensity expressed through the percentage of the working load expressed by the number of RM varied between 40 to 65% of 8 RM [39,44], from 40 to 50% of 8 RM [45], and from 60 to 90% of 1 RM [47]. Different purposes, different available material resources and/or individual characteristics may be some of the reasons to justify such a range of training intensities used in the different studies, not fulfilling the usual recommendations suggested by the ACSM [48]—75–80% 1 RM. Despite the reported use of different intensities, in general, the ACSM guidelines [48] were applied and all studies reported a positive effect (see ST results section).

The application of the training progression general principle was common to several studies [40,41,43,45,46,47], with an increase in intensity throughout the intervention period, regardless of the type of material/equipment used (whether the programs used weight machines, free weights, rubber bands, or other materials). The progression of the intensity increased gradually, depending on the number of training weeks or individual performance, either by increasing the percentage of the training load (by increasing the weight in the ankle shin guards), or simply by increasing the number of series and/or repetitions to perform during the training session, as recommended by the ACSM [48,50].

### 4.7. Exercises

According to the studies evaluated [40,43,45,46,47], most ST programs included exercises targeting the main muscle groups in each session. Although the studies analyzed use different types of equipment (weight training machines, using resistance elastics, and/or ankle weights), all of them show intentionality to use ST exercises aiming to request the main muscle group [50]. It should be noted that only two studies used a period of adaptation and familiarization to the prescribed exercises [52,53], which is important to eliminate the fear of using new materials, movement perception, and to ensure high quality results. The most common exercises used in the ST programs are the leg flexion and leg extension exercises (hamstrings and quadriceps), the abdominals in their different variants (abdomen muscles), the chest press (pectoral major), the low row or the lat pull down (latissimus dorsi), flexion of the forearm (biceps), an extension of the forearm (triceps), and elevation, abduction, or shoulder press (deltoids). When prescribing six to eight exercises, ST programs were in accordance with the recommendations provided by ACSM [48], however, in some studies, this was not the case [40,42]. In the majority of the studies, selected exercises tended to be simple and easy to be performed, with special attention and reinforcement in the instruction, demonstration, and familiarization [48,52,53]. Some individuals may experience difficulties in controlling movement, particularly in the eccentric phase, and thus it was suggested to use machines that help to better control them (for example, a chest press device rather than the bench press). Additionally, machine exercises are preferable to avoid some type of injury, for presenting a smaller range of motion; however, there were some exceptions such as the biceps curl, seen as working well in participants with ID [50].

### 4.8. ST Programs Outcomes

Some studies have shown significant positive effects on muscle strength in lower limbs [46], in upper and lower limbs [47], and in handgrip strength (*p* < 0.0001) [43]. These results are very encouraging for further studies with this population and to implement in clinical practice. A greater capacity to generate strength by the muscles (lower and upper limbs) may become an essential tool to insert these patients in professional activities due to the increase in their physical capacities [46].

Other studies found an increase in fat-free mass and a reduction in fat mass [42,43,44]. Depending on the aims and evaluation methods used, some studies reported a reduction in the waist circumference [44] of the BMI [43] and an improvement in balance [40].

An increase in the concentration of salivary immunoglobulin, testosterone levels, plasma leptin levels, TNF-α, and IL-6 was also found. Specifically, Fornieles et al. [39] showed thar resistance training program of 12 weeks increased concentration of salivary immunoglobulin (*p* = 0.0120), testosterone levels (*p* = 0.0088) and task performance (*p* = 0.0141). This study highlights the benefits of ST, as this increase in salivary IgA levels can prevent respiratory tract infections in individuals with DS [54]. This study also shows an improvement in the anabolic status of DS patients after the ST program, as cortisol levels remain unchanged and there was an increase in salivary testosterone. It was also found that the improvement of task performance is of great interest to this population. Having improved levels of muscle strength may allow this population to perform a greater number of activities and continue to exercise, thus reducing the risk of secondary consequences for their health [55,56]. Additionally, improvements in the response to systemic inflammation, in the antioxidant defense system, and a reduction in oxidative damage were also reported [39,44,45].

Dynamic balance as a parameter of functional capacity is also limited in individuals with ID. Even so, two studies reported improvements with ST, particularly in exercises focused to improve strength and power of the lower limbs [40,46]. These improvements were also associated with improvements in gait speed and balance [46]. Other non-randomized controlled studies have shown interesting results through the implementation of ST programs in individuals with ID, namely, cognitive effects such as positive changes in working memory, short-term memory, vocabulary knowledge, and reasoning ability [57], and improved flexibility [58] and performance in daily life activities [59]. There is an urgent need for randomized procedures to assess the benefits of these variables and aerobic capacity. Several studies have found significant differences in body composition parameters after the strength training program, namely a reduction in the BMI (*p* < 0.0001) and the skin fold of the calf (*p* = 0.008) [43]; a decreased in waist circumference (*p* = 0.0416) and increase in the fat-free mass (*p* = 0.011) [44]; and an increased in the lean mass (*p* = 0.008) and reduction in the fat percentage (*p* = 0.036) [43]. Since being overweight and obesity are associated with poor health and quality of life, these results show that strength training is a good intervention to reduce these values.

With the implementation of ST programs, despite the different prescriptions, positive results were verified in terms of the aims defined in all studies, which shows how training variables, techniques, and methods (for example, training frequency and exercise selection volume, training load and repetitions, and others), can be manipulated to optimize training response. The different results presented in Table 4 and of the non-randomized controlled studies [57,58,59] are demonstrated due to the different aims and evaluation methods used in each study, being an added value the application of ST, since it can have this wide range of benefits. All these results are important in terms of promoting the quality of life of individuals with ID, related to the conceptual model of Schalock et al. [60], a construct divided into three dimensions: (i) independence; (ii) social participation; (iii) well-being.

Some studies included in the systematic review have some limitations, which may limit the magnitude of the results. Future studies should take them into account when implementing ST programs: (i) short-term studies [39,44,47]; (ii) no follow up to determine whether the positive effects were maintained [39,44,45,47]; (iii) small sample size [41]; (iv) sample size only boys [41]; (v) exclusion of children with severe and profound ID [41]; (vi) a small number of professionals to supervise the exercises produced in the experimental protocol [42]; (vii) lack of more accurate measurements to evaluate; and (viii) a small number of outcomes [47].

At the same time, future studies are suggested to apply the assessment of body composition variables by electrical bioimpedance, such as the phase angle, since it has been considered a relevant marker of health status [61]. Moreover, a higher phase angle value is positively associated with a higher quality of cell membranes [62], while a lower value is associated with deterioration, which can compromise all cell functions [62]. Thus, phase angle assessment would be helpful to understand if there are adaptations with ST in this variable that has a strong correlation with cell health and integrity, being an excellent indicator of the capacity of the cell membrane to retain liquids, fluids, and nutrients in the population with ID.

The fact that ID is a multisystem and complex disorder characterized by the presence of delays or deficits in the development of adaptive behavior comprising conceptual, social, and motor skills may support a possible explanation why results could differ between studies and studies without the RCT method. The effects of the interventions in the various studies appear similar to those that have been reported following resistance training in the average healthy and intellectually disabled population [3,22,25,30,33]. However, some variability has also been reported in other resistance training studies and those examining other exercise modalities [20,29,46,47,57]. Inherent within any measurement are both technical error and random within-subject variation [63]. Studies included in this systematic review have shown that, although using different training intensities, it was possible to identify improvements in the variables under study; however, it is noted that, before starting the training program, it is necessary to carry out strength assessments, either through RM tests, using devices, or isokinetic, as recommended by ACSM [48], to determine the correct intensity to be used. During this strength assessment process, as during training, RM tests using free weights, push-ups, and pull-ups should be avoided [48] to prevent any type of injury. Moreover, familiarization with the assessment procedures, a practical demonstration of execution, simple instructions, constant supervision, and verbal and visual reinforcement are necessary [48,53] for greater success in the didactic-pedagogical process.

This systematic review analyzed the effects of ST in individuals with intellectual disability, aiming to be a reference guidelines tool for researchers and professionals of PE. The analyzed studies show characteristics and recommendations that professionals can follow when implementing an ST program to promote benefits and positive outcomes, namely, the maintenance of/increase in physical fitness, quality of life, and health, thus decreasing the risk of developing chronic diseases, being the strong aspect of this systematic review. Therefore, it is essential to implement this type of ST program, incorporated into the weekly routine of this population, which, when associated with an appropriate lifestyle, causes a set of adaptations and benefits and, ultimately, can promote a decrease in clinical expenses, an increase in healthy aging, and better health.

It is recommended to increase the implementation of ST programs in the target population, expanding the knowledge in terms of the methods, structure, and duration used, so that professionals can prescribe adapted and effective ST programs. At the same time, it is important that the exercise professionals have an in-depth knowledge of the individual, their comorbidities, limitations, and preferences, before prescribing and starting a PE program [50].

Despite the relevance of the selected clinical trials for the preparation of this systematic review, some limitations can be observed: (1) the diversified intervention methodology, involving different strengths, intensities, volumes, and weekly training exercise programs; (2) unclear descriptions of the process of randomization and allocation of people with ID in the groups; (3) loss of follow-up; (4) different evaluation methodologies, as well as the results, not allowing a further discussion as well as a meta-analysis about the effects produced by the several ST programs applied; (5) the level of ID was not mentioned in all studies included, which limits the generalization of the results and suggests that future studies should mention such specificity.

## 5. Conclusions

According to the studies included in this systematic review, it can be concluded that the ST program interventions (particularly when not combined with other exercises) for individuals with ID are effective in positive muscle strength and present positive outcomes that contribute to the improvement of functional capacity. However, the limited number of studies and the low study quality scores indicate the potential risk of bias, which limits the interpretation of the findings and warrants further investigation. Further studies on ST are needed to better analyze the training program characteristics and their effects on individuals with ID.

From the analyzed studies, the following aspects were considered transversal in the ST prescription:

(a) Duration of 12 weeks for mesocycles;
(b) Most are applied at least three times per week;
(c) Duration of 45 to 60 min per session;
(d) Six to seven exercises recruiting the main muscle groups, avoiding free weights, such as chest press, low row or lat pull-down, elevation, abduction or shoulder press, and abdominals due to their different variants, flexion of the forearm, the extension of the forearm, and leg extension/leg curl/leg press;
(e) For each exercise: two or three sets with 6 to 12 repetitions per exercise or maximum. The review of the intensity is essential for the overload process, which can be carried out in the ways mentioned above.
(f) Testing and assessment protocols used in ST programs should be individualized for adults with ID to accommodate their characteristics and should be implemented under conditions similar to those experienced during the training regimen.
(g) It is important to implement familiarization sessions before carrying out muscular strength testing or initiating an ST program to ensure safety, accuracy, and effectiveness of the program for adults with ID.

There are several benefits of ST in the individual with ID: (i) increase in the strength of the lower and upper limbs, of the fat-free mass, balance, concentration of salivary immunoglobulin, testosterone levels, plasma leptin levels, factors of tumor necrosis alpha, and interleukins 6; (ii) reduction in waist circumference, BMI, fat mass and oxidative damage; (iii) improved response to systemic inflammation and antioxidant defense system.

We hope this systematic review will be a reference tool for researchers and exercise professionals when prescribing and implementing ST programs.

## Figures and Tables

**Table 1:** Research strategy.

Search Number	Research Content
1	(“mental retardation” OR “intellectual disability” OR “intellectual disabilities” OR “down syndrome”) AND (“resistance training” OR “strength training” OR “neuromuscular training” OR “resistance exercise” OR “strength exercise” OR “neuromuscular exercise ”)

**Table 2:** Assessment of the quality of the articles, using the PEDro scale, as well as their total score.

Author (Year)	PEDro Scale											Total
	1	2	3	4	5	6	7	8	9	10	11	
Fornieles et al. [39]	s	1	0	1	-	-	0	1	1	1	1	6
Ghaeeni et al. [40]	s	1	0	1	-	-	0	1	1	1	1	6
Kachouri. et al. [41]	s	1	0	1	-	-	0	1	1	1	1	6
Neto et al. [42]	s	1	0	1	-	-	0	1	1	1	1	6
Ortiz-Ortiz et al. [43]	s	1	0	1	-	-	0	1	1	1	1	6
Rosety-Rodriguez. et al. [44]	s	1	0	1	-	-	1	1	1	1	1	7
Rosety-Rodriguez et al. [45]	s	1	1	1	-	-	0	1	1	1	1	7
Shields and Taylor [46]	s	1	1	1	-	-	1	1	1	1	1	8
Shields et al. [47]	s	1	1	1	-	-	1	1	1	1	1	8

Notes: Item 1, not scoreable; Items 5 and 6, no applicability.

**Table 3:** Characteristics of the strength training programs of the nine studies.

Author, Year, Country	Aims	Participants	Study Design	Assessment Tools/Techniques
Fornieles et al. [39] Spain	Influence of ST on salivary immunoglobulin A levels and hormone profile in sedentary DS adults.	N = 40 ♂; age: 23.7 ± 3.1; DS (IQ: 60–69); randomized groups: GE: N = 24 | CG: N = 16).	Prospective cohort.	8 RM test (exercises: bicep curl; leg extension; seated row; leg curl; triceps extension; leg press); saliva samples—analysis of immunoglobulin, testosterone, and cortisol (ELISA kits); box stacking test (ACSM, 2013; Smail and Horvat, 2006).
Ghaeeni et al. [40] Iran	Effect of 8 weeks core stability training on static balance of DS children.	N = 16; age: 9.7 ± 1.7 y; DS; randomized groups: GE: N = 8 | CG: N = 8.	Prospective cohort.	Static balance—stork test (Rahmani and Shahrokhi, 2011).
Kachouri et al. [41] Tunisia	Effect of a combined strength and proprioception training program on muscle strength and postural balance in children with ID.	N = 20 ♂; age: 11.5 ± 1; ID (IQ: 50–70); randomized groups: GE: N = 10 | CG: N = 10.	Prospective cohort.	Maximum voluntary contraction—quadríceps (dynamometer or manual muscle testing—Bohannon, 2005; Brinkmann, Andres, Medoza, and Sanjak, 1997); centre of pressure—static stabilometric platform (PostureWin©, Techno Concept^®^, Cereste, France; 12-bits A/D conversion).
Neto et al. [42] Brazil	Effects of ST on body composition.	N = 15 (♂ = 11; ♀ = 4); age: 22.1 ± 7.5; DS; randomized groups: GE: ♂ = 6; ♀ = 2 CG: ♂ = 5; ♀ = 2.	Prospective cohort.	Body mass—electronic scale model Filizola (Indústria Filizola S/A, São Paulo, Brazil); percentage of fat—seven thoracic, axillary, tricipital, subscapular, abdominal, supra-iliac, and thigh skinfolds (adipometer); fat mass calculated using the formula: body mass × percentage of fat/100; lean mass calculated using the formula: body mass − fat mass.
Ortiz-Ortiz et al. [43] Mexico	Effect of a physical fitness program on body composition and isometric strength in DS children.	N = 22; age: 11.8 ± 1.9 y; DS; randomized groups: GE: N = 13 | CG: N = 9.	Prospective cohort.	Body weight—Tanita^®^ InnerScan (BC-533, Tanita Corporation of America, Inc., Clearbrook, IL, USA); BMI = weight ÷ (height^2^); percentage of fat—subcutaneous triceps and calf sites; isometric strength—manual dynamometer—(dominant hand) MSD, (model SH5001, Düsseldorf, Germany).
Rosety-Rodriguez et al. [44] Spain	Effect of ST on low-grade systemic inflammation in DS adults.	N = 40 ♂; age: 23.7 ± 3.1; DS (IQ: 60–69); randomized groups: GE: N = 24 | CG: N = 16.	Prospective cohort.	Blood samples—plasma levels of leptin, adiponectin, interleukin-6 and TNF-α (ELISA kits); C-reactive protein—nephelometry; fat-free mass percentage—bio impedance (Tanita TBF521, Tanita Corporation of America, Inc., Clearbrook, IL, USA); waist circumference—anthropometric tape; time up-and-go test (Rikli and Jones, 1999).
Rosety-Rodriguez et al. [45] Spain	Effect of ST on antioxidant defence system in sedentary DS.	N = 36 ♂; age: 28.1 ± 3.3; DS (mild ID–IQ: 60–69); randomized groups: GE: N = 18 | CG: N = 18.	Prospective cohort.	8 RM test (exercises: arm curl, leg extension, leg curl, low stroke, triceps extension and leg press); blood samples—puncture of the antecubital vein; maximum force—manual dynamometer JAMAR (Bolingbrook, IL, USA); peak torque of flexion and extension of the of the knees—isokinetic dynamometer at 90°/s -Technogym-REV 9000 (Technogym Spa, Gambettola, Italy); total antioxidant status of plasma—spectrophotometrically, Hitachi 902 Autoanalyzer (Roche, Alameda, CA, USA) by commercial kits (Randox, Crumlin, UK); reduced glutathione level after reaction with DTNB [(5,5-dithio-bis (2-nitrobenzoic acid)]; superoxide dismutase activity—xanthine oxidase-cytochrome c method; glutathione reductase activity; plasma ascorbate and α-tocopherol—reverse phase high-performance liquid chromatography.
Shields and Taylor [46] Australia	Effects of ST on the ability to produce muscle strength and physical fitness.	N = 23 (♂ = 17; ♀ = 6); age: 15.6 ± 1.6; DS (mild to moderate ID); random groups: GE: N = 11 | CG: N = 12.	Prospective cohort.	1 RM test (chest and leg press); timed Up and Go test (Rikli and Jones, 1999); down stairs test (Zaino et al., 2004); grocery shelving task (Hill et al., 2004).
Shields et al. [47] Australia	Effects of ST in adolescents and young DS adults.	N = 68 (♂ = 38; ♀ = 30); age: 17.9 ± 2.6; DS (mild to moderate ID); random groups: GE: N = 34 | CG: N = 34.	Prospective cohort.	Box stacking test (ACSM, 2013); weighted pail carry test (ACSM, 2013); 1 RM test (chest and leg press).

Abbreviations: BMI, body mass index; CG, control group; DS, Down Syndrome; GE, exercise group; ID, intellectual disability; IQ, intelligence quotient; min, minutes; N, number of participants; s, seconds; RM, maximum repetition; ST, strength training; ♂, male gender; ♀, female gender.

**Table 4:** Characteristics of the strength training protocols from the nine studies.

Author, Year	Program Duration, Frequency, Session Duration	Exercise Protocol	Results
Fornieles et al. [39]	12 weeks; 3 × week; session duration ND.	Exercises: arm curl; leg extension; seated row; leg curl; triceps extension; leg press; intensity: 40 a 65% 8 RM; 2 sets; 6 to 10 rep; 90 sec rest.	Increased concentration of salivary immunoglobulin (*p* = 0.0120), testosterone levels (*p* = 0.0088) and job performance (*p* = 0.0141).
Ghaeeni et al. [40]	8 weeks; 3 × week; 45 a 60 min/session.	Abdominal workout; 3 to 4 exercises per session; 3 to 6 sets; 10 to 20 rep.	Improvement of static balance (*p* = 0.0001).
Kachouri et al. [41] Tunisia	8 weeks; 3 × week; 45 a 60 min/session.	All exercises were performed in two surfaces, firm and foam; exercises included air squat, squat jumps, straight sit ups, power sit up, flutter kicks, two-foot ankle hop, single-foot side-to side ankle hop, tuck jump with knees up, standing long jump, double leg hops, single leg hops, standing on one-foot, lateral jump with both feet, lateral jump with one foot, running up the stairs with one foot and running up the stairs with both feet. 3 to 5 sets; 15 to 20 rep.	Improves postural balance.
Neto et al. [42]	12 weeks; 3 × week; 60 min/session.	Exercises: chest press, squat, shoulders, leg curl, one-sided stroke, heel lift, bicipital curl, tricipital French e abdominal crunch; 3 sets; 8 to 12 rep; 30 to 60 sec rest.	Increased lean mass (*p* = 0.008) and reduced fat percentage (*p* = 0.036).
Ortiz-Ortiz et al. [43]	16 weeks; 5 × week; 55 min/session.	Circuit exercises using weight disks, rubber bands, dumbbells, medical balls and shin guards with weights—biceps curl, triceps extension, chest press, and handgrip with different degrees of tension.	Reduction in the BMI (*p* < 0.0001) and the skin fold of the twin (*p* = 0.008); increased isometric strength (*p* < 0.0001).
Rosety-Rodriguez et al. [44]	12 weeks; 3 × week; session duration ND.	Exercises included arm curl, leg extension, seated row, leg curl, triceps extension, and leg press; 40 to 65% of 8 RM; 2 sets; 6 to 10 rep.	Plasma levels of leptin (*p* < 0.05), TNF-α (*p* < 0.05) and IL-6 (*p* < 0.05) and waist circumference decreased (*p* = 0.0416); increase in fat-free mass (*p* = 0.011); improved response to systemic inflammation.
Rosety-Rodriguez et al. [45]	12 weeks; 3 × week; Session duration ND.	Exercises included arm curl, leg extension, seated row, leg curl, triceps extension, and leg press; 40 to 50% of 8 RM; 2 sets; 8 to 10 rep; 90s rest.	Improvement of the antioxidant defense system; reduction in markers of oxidative damage.
Shields and Taylor [46]	10 weeks; 2 × week; session duration ND.	Exercises: lat pull-down, seated chest press, seated row, seated leg press, knee extension, calf raise; 3 sets; 12 rep or until fatigue; 2 min rest between exercises.	Improvement in muscle strength of the lower limbs (mean difference 36 kg, 95% CI 15 to 58).
Shields et al. [47]	10 weeks; 2 × week; 60 min/session.	Exercises: lat pull-down, seated chest press, seated row, seated leg press, knee extension, seated calf raise; 3 sets; 12 rep; 60 to 80% RM; 2 min rest between exercises.	Improvement in muscle strength of the lower 1imbs (mean difference 25 kg, 95% CI 8 to 42) and upper limbs 1 (mean difference 7 kg, 95% CI 3 to 11).

Abbreviations: min, minutes; ND, not defined; rep, repetition; RM, maximum repetition; s, second.

## Data Availability

Additional data are available upon request to the author for correspondence.
